# Supplementary material for: A comprehensive assessment of demographic, environmental, and host genetic associations with gut microbiome diversity in healthy individuals
Source: Microbiome. 2019 Sep 13;7:130. doi: 10.1186/s40168-019-0747-x (PMC6744716; doi:10.1186/s40168-019-0747-x)
Supplement: Supplementary file 1 — Figure S1. Raw and transformed distributions and violin plots of α-diversity phenotypes. Figure S2. Multidimensional scaling plots of Jaccard and Unifrac distance matrices. Figure S3. Number and overlap of non-genetic variables associated with α-diversity phenotypes. Figure S4. Correlations of age and ALT levels with Simpson’s diversity index. Figure S5. Number and overlap of non-genetic variables associated with ß-diversity matrices. Figure S6. Data plots showing identified correlations of non-genetic variables with three individual taxa. Figure S7. Manhattan plots for α-diversity metrics: richness, Chao1 and ACE. Figure S8. Manhattan plots for ß-diversity matrices: Jaccard and Unifrac. Figure S9. QQ plots and lambda values of GWAS of α-diversity phenotypes. Figure S10. QQ plots and lambda values of GWAS of β-diversity indexes. Figure S11.Manhattan plot of HLA and KIR association results with all phenotypes. Figure S12. PCoA plot of samples obtained from different sequencing batches. Figure S13. PCA plot of the genetic matrix data of MI donors. (DOCX 2702 kb) [file 40168_2019_747_MOESM1_ESM.docx]

**
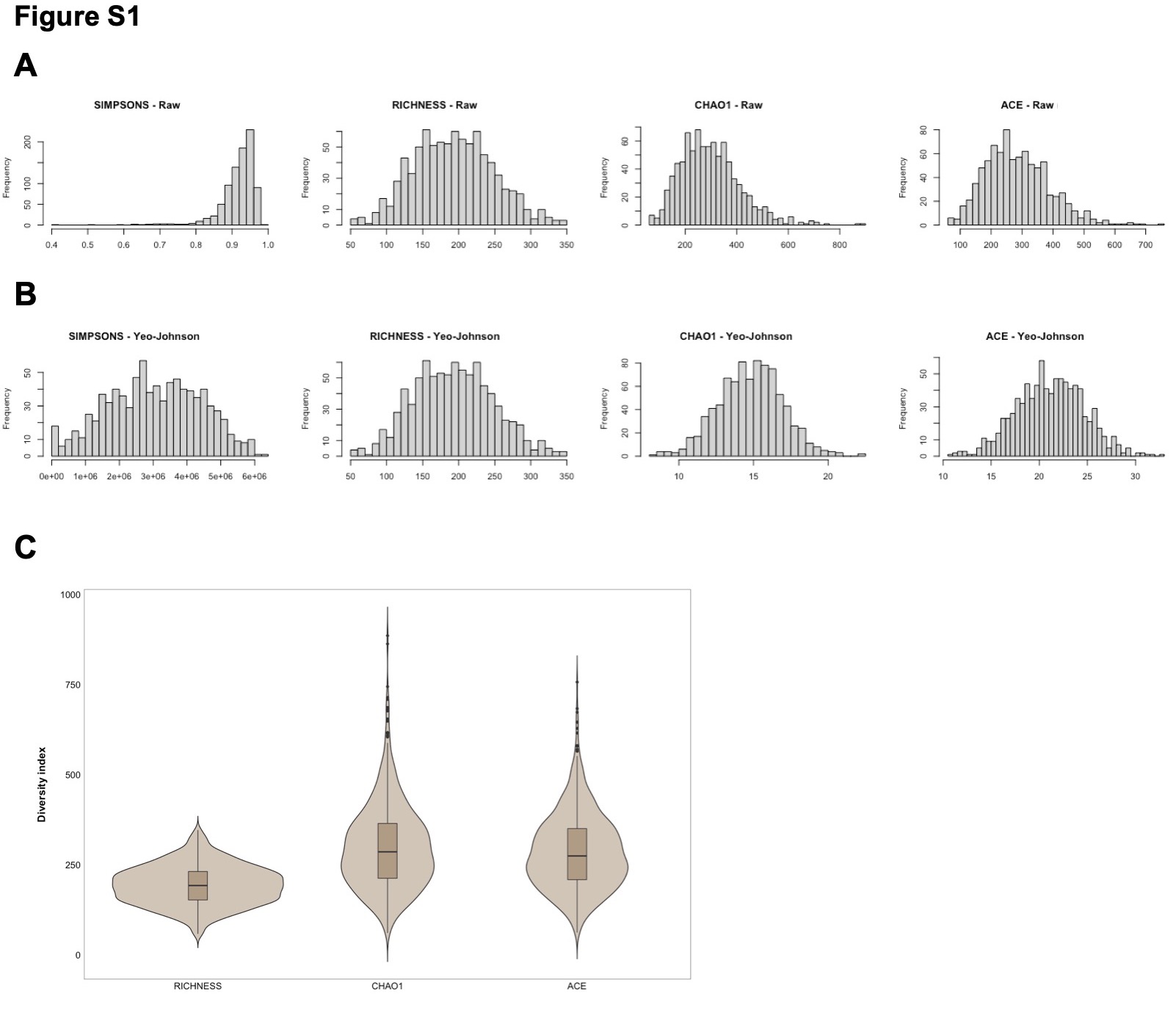
**

**Figure S1.** Distributions of four (A) raw and (B) Yeo-Johnson transformed α-diversity metrics. (C) Violin plots of distributions of α-diversity metrics – Richness, Chao1 and ACE.


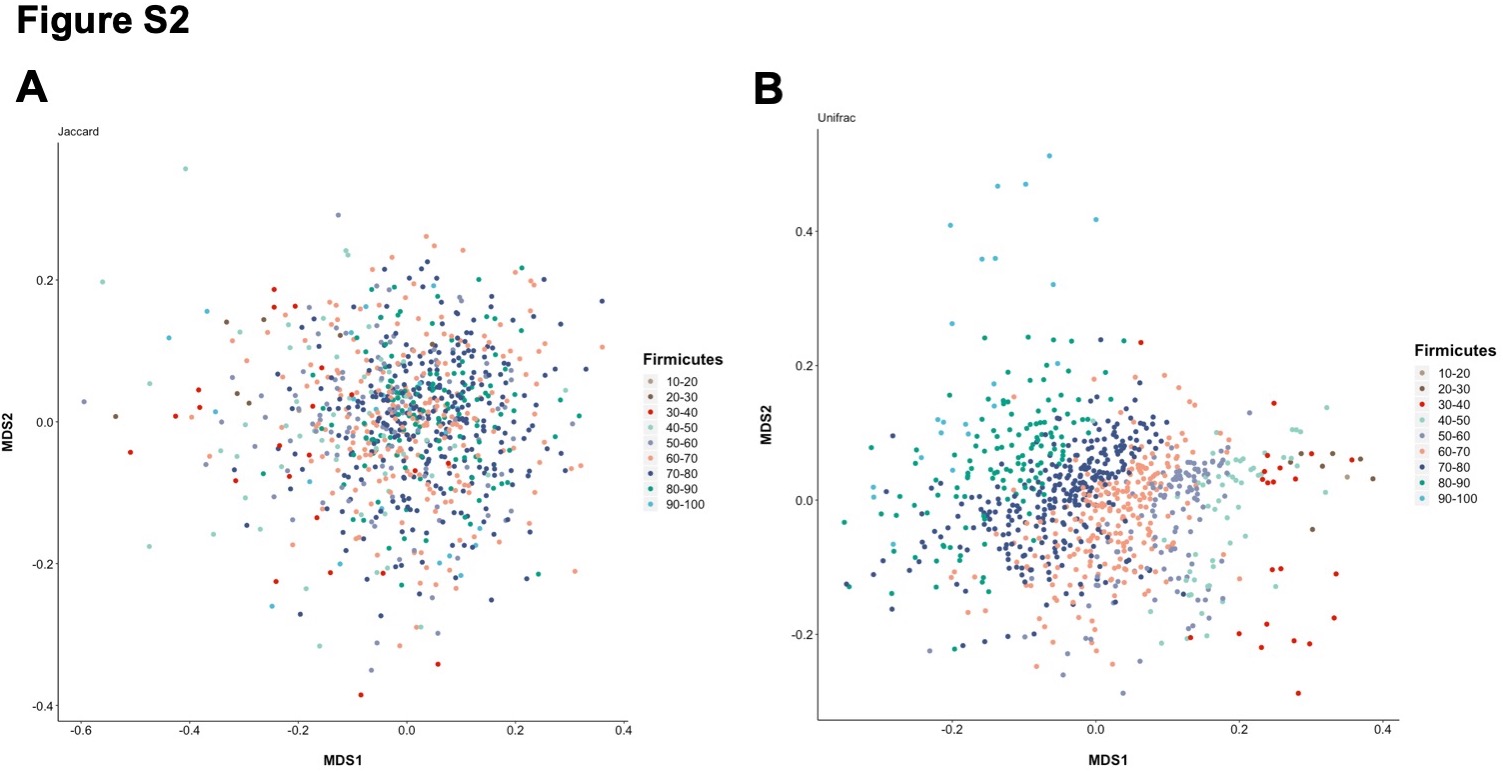


**Figure S2.** Multidimensional scaling plots of Jaccard (A) and Unifrac (B) distances with samples colored according to the relative abundance of *Firmicutes*.


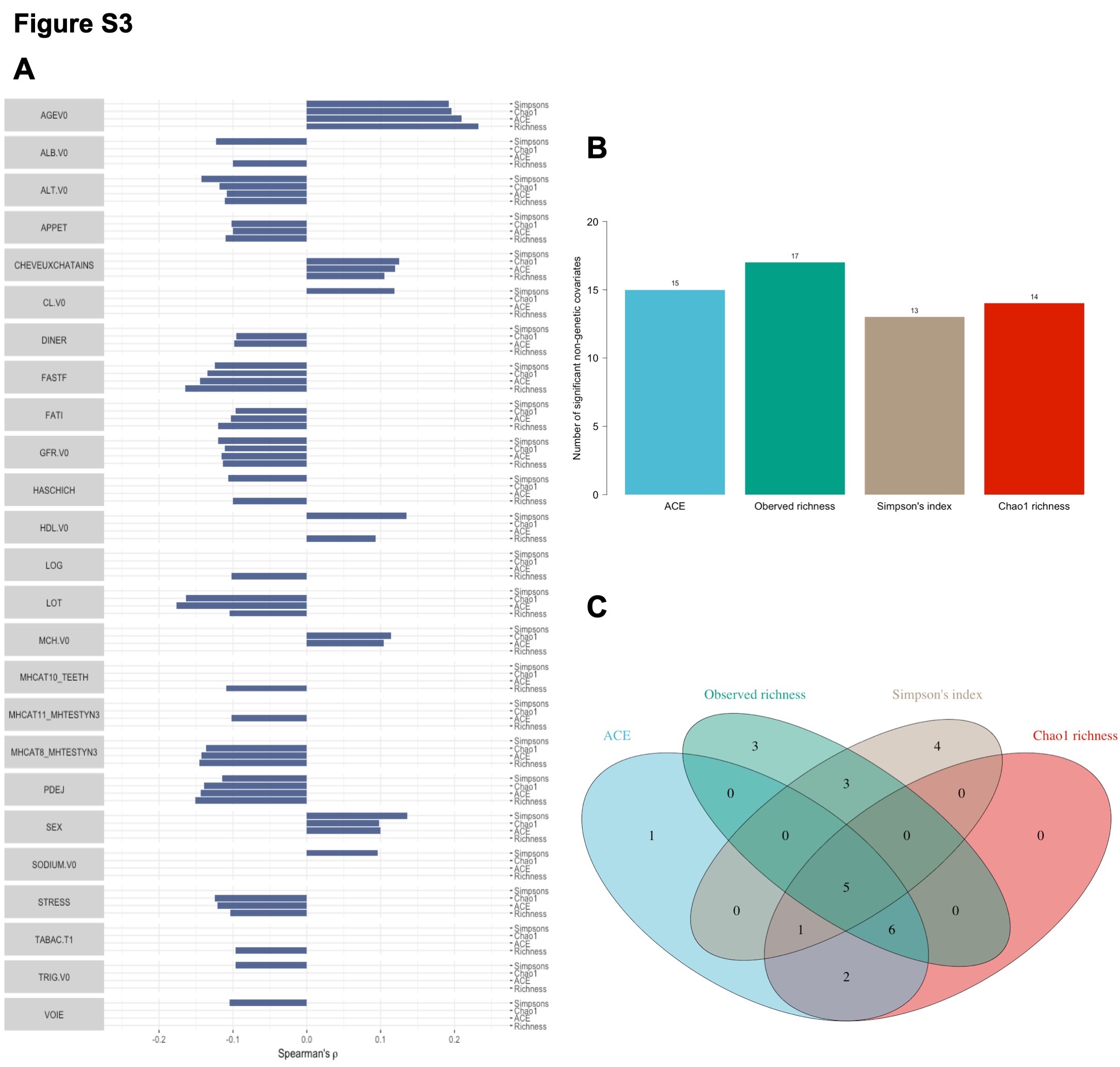


**Figure S3.** (A) 25 non-genetic variables are significantly associated with at least one α-diversity metric in univariate tests (FDR < 0.05). The length of the blue bars shows the Spearman’s ρ value of the significant associations; (B) Number of significant associations between non-genetic covariates and each of the four α-diversity metrics in univariate tests; (C) Overlap of the significant associations between α-diversity metrics.

A full description of the non-genetic variables is available in Additional File 2: Table S1.

**
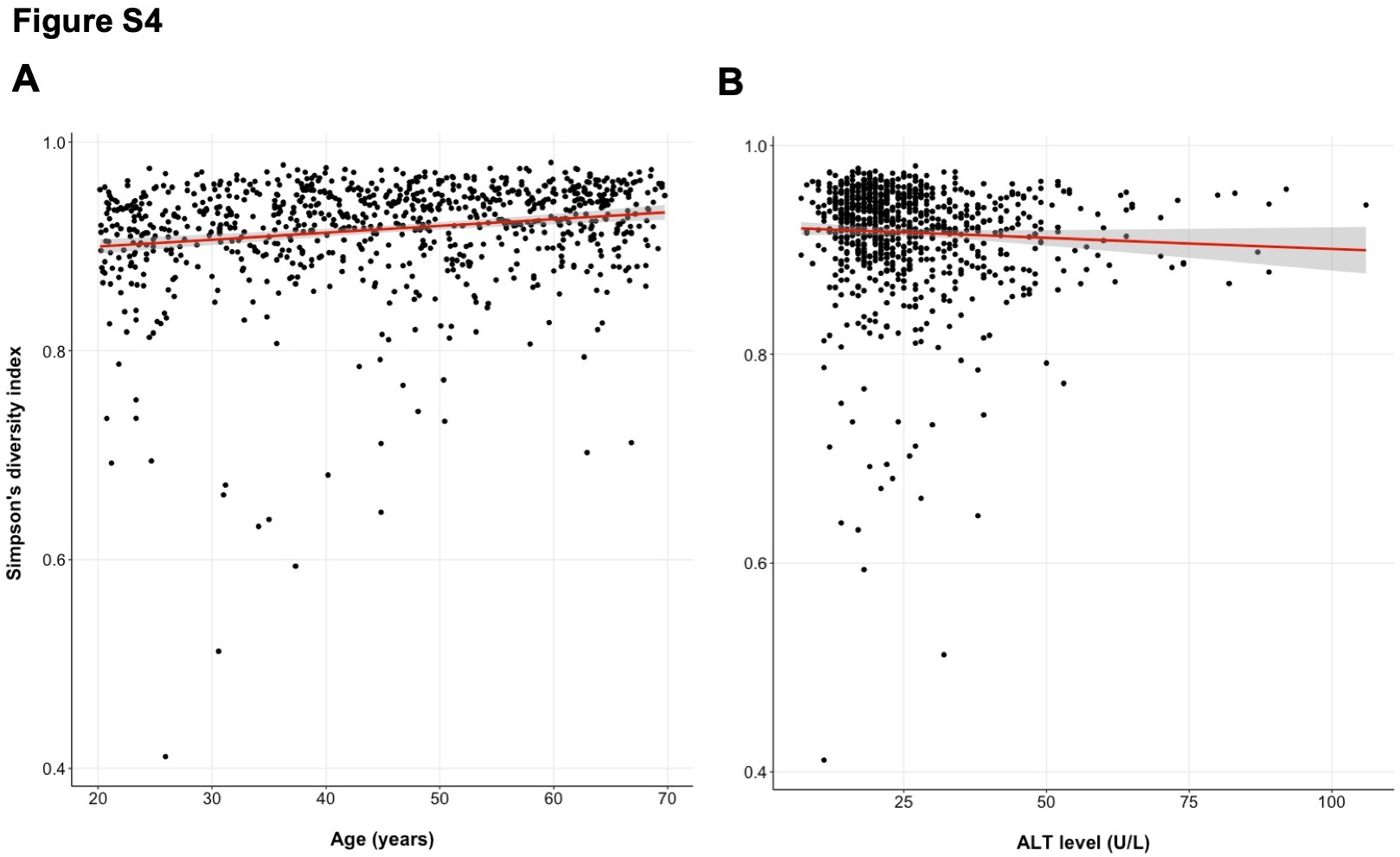
**

**Figure S4.** Scatter plots and the line of the best fit of Simpson’s diversity index (representative

α-diversity metric) with (A) age and (B) ALT level.

**
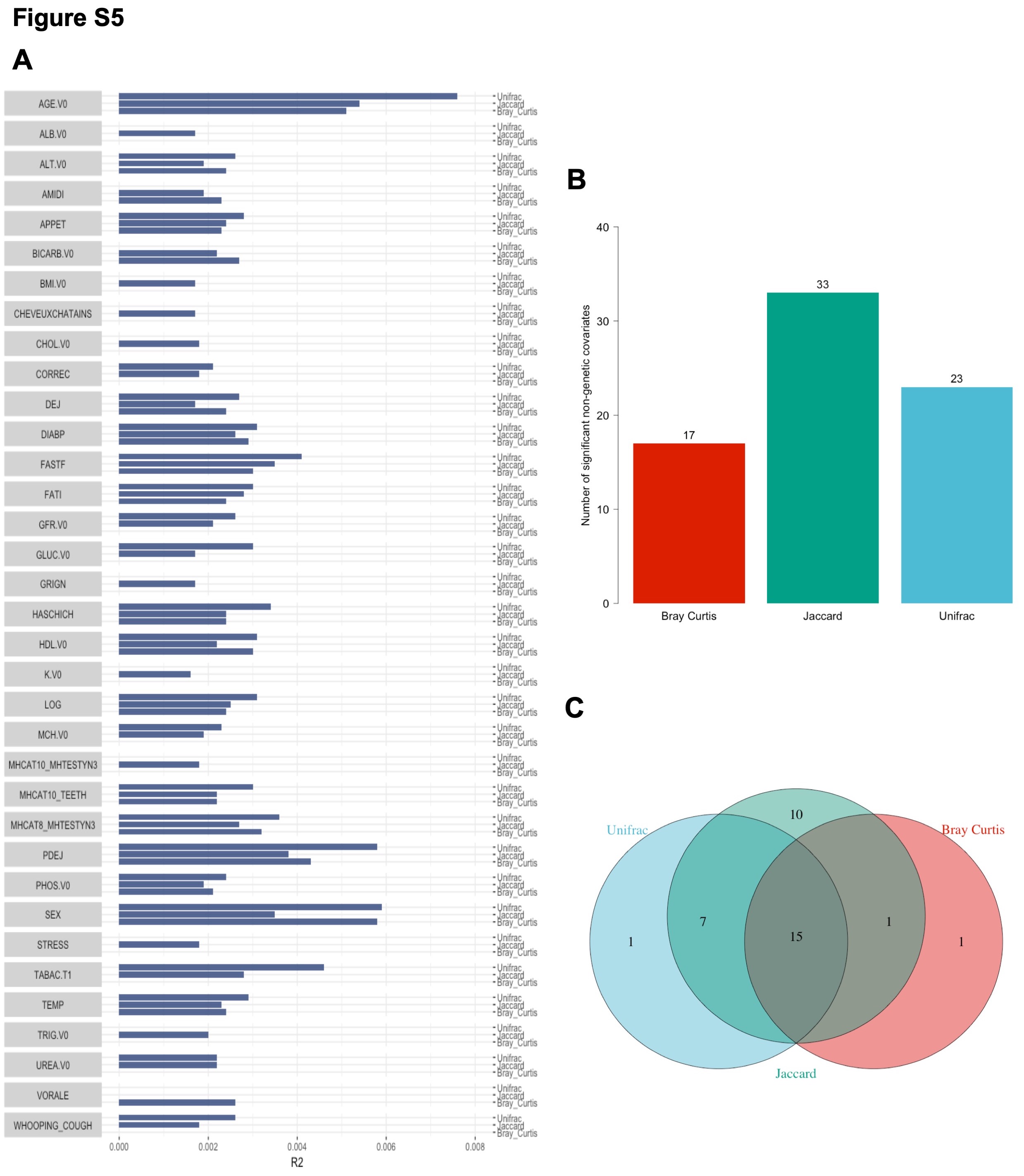
Figure S5. (**A) 35 non-genetic variables are significantly associated with at least one β-diversity metric in univariate tests (FDR < 0.05). The length of the blue bars shows the R^2^ value of the significant associations; (B) Number of significant associations between non-genetic covariates and each of the three β-diversity metrics in univariate tests; (C) Overlap of the significant associations between β-diversity metrics.

A full description of the non-genetic variables is available in Additional File 2: Table S1.


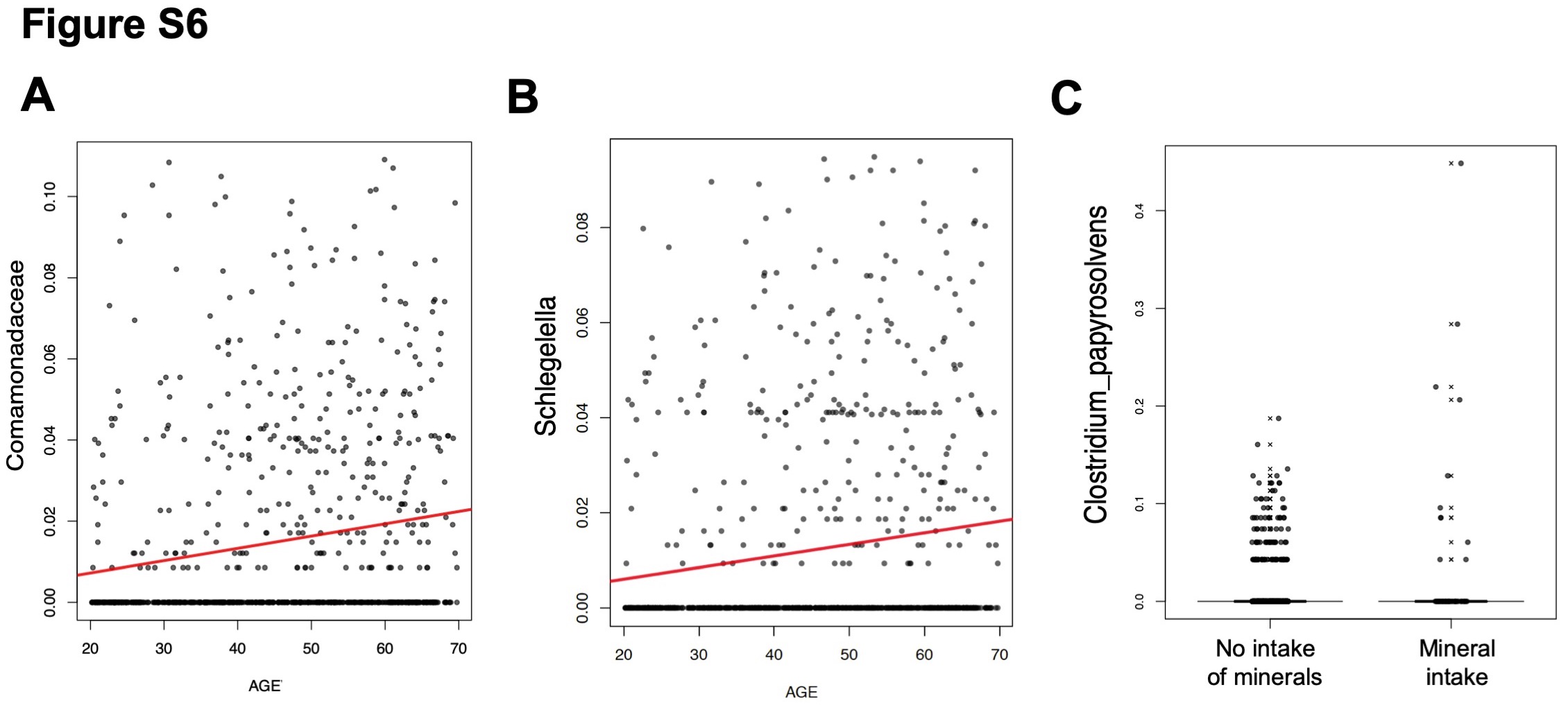


**Figure S6.** Data plots indicating association between age and *Comamonadaceae* (A) or

*Schlegelella* (B); and between consumption of minerals and *Clostridium papyrosolvens* (C).

**
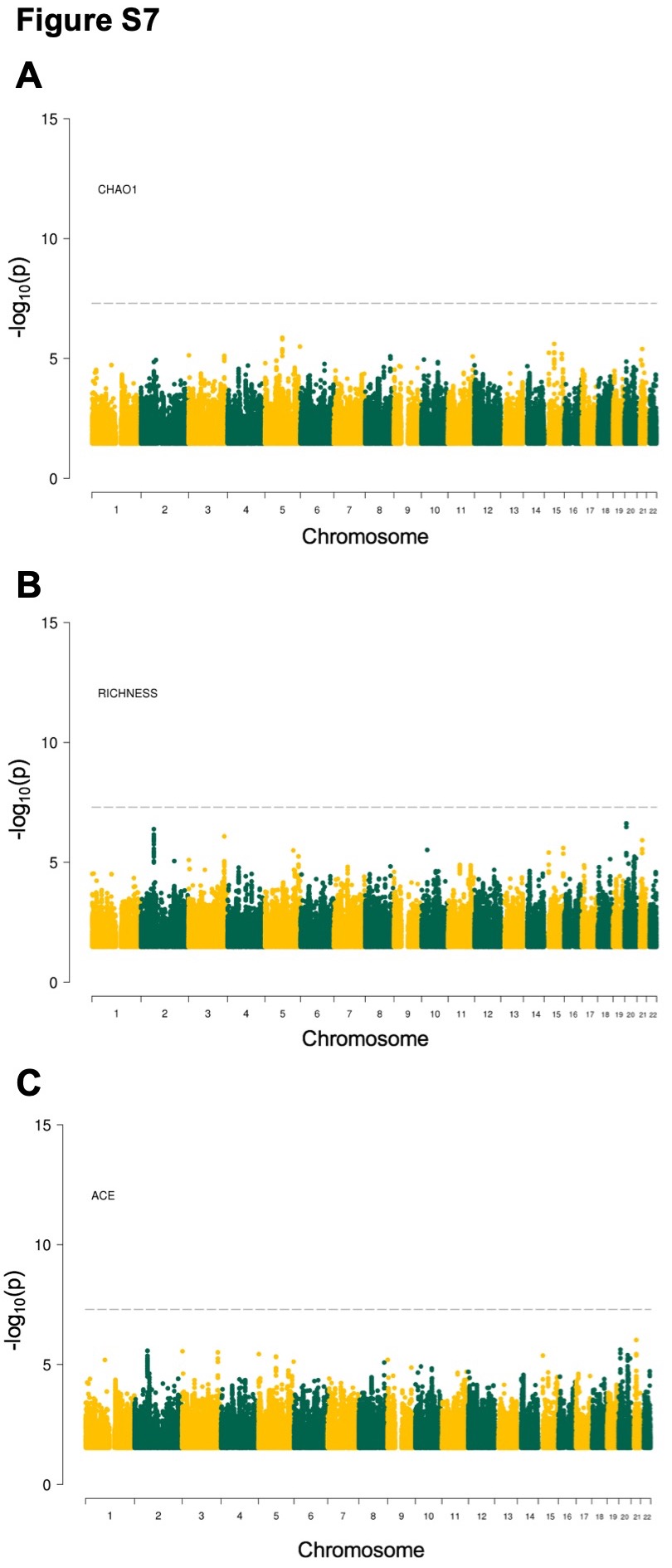
**

**Figure S7.** Manhattan plots showing the results of GWAS of (A) Chao1 index, (B) richness and (C) ACE. The dashed horizontal line denotes genome-wide significance threshold (P_α-threshold_ < 1.25 x 10^-8^).

**
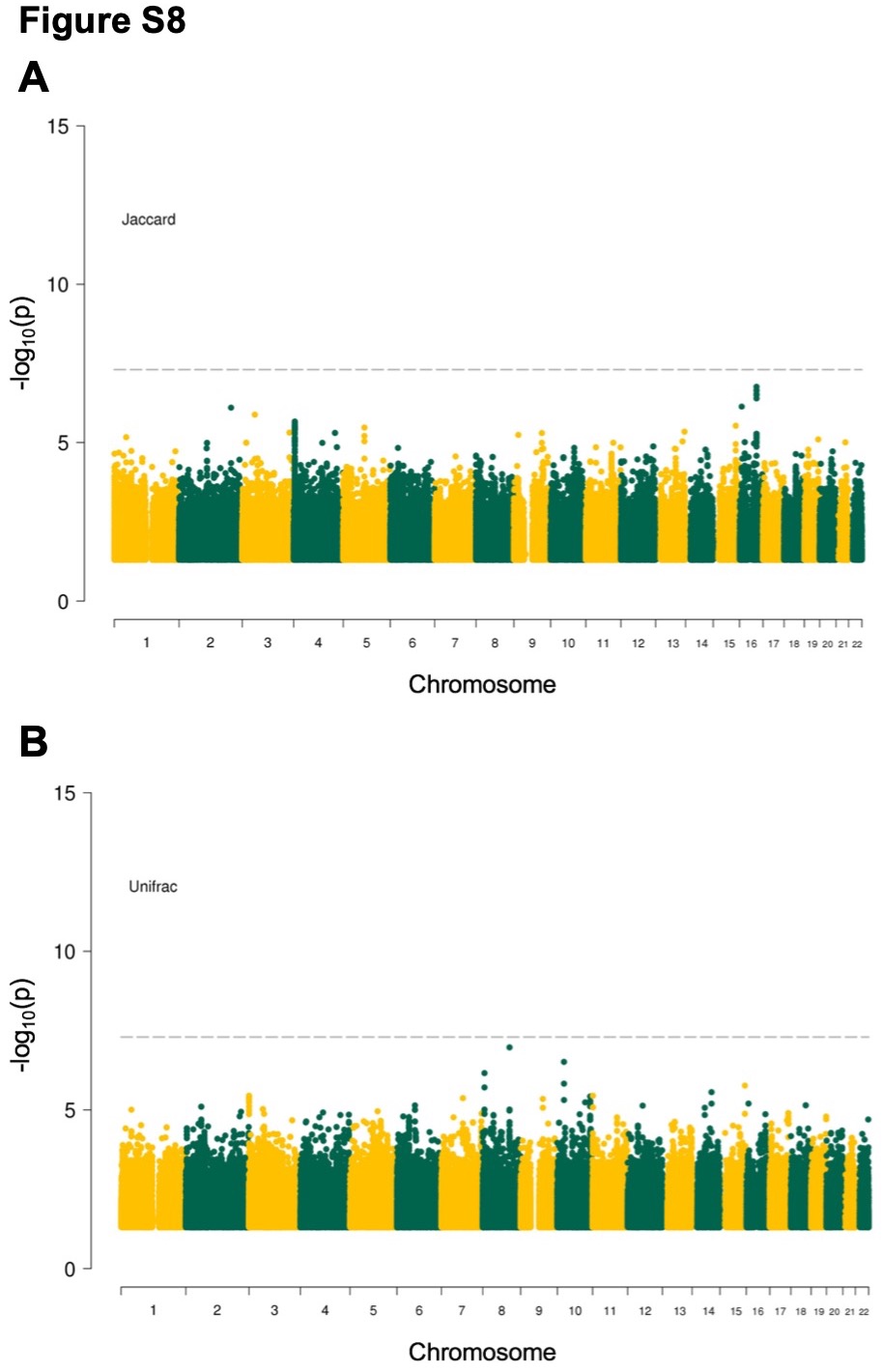
**

**Figure S8.** Manhattan plots showing the results of GWAS of (A) Jaccard and (B) Unifrac distances. The dashed horizontal line denotes genome-wide significance threshold (P_β-threshold_ < 1.67 x 10^-8^).

**
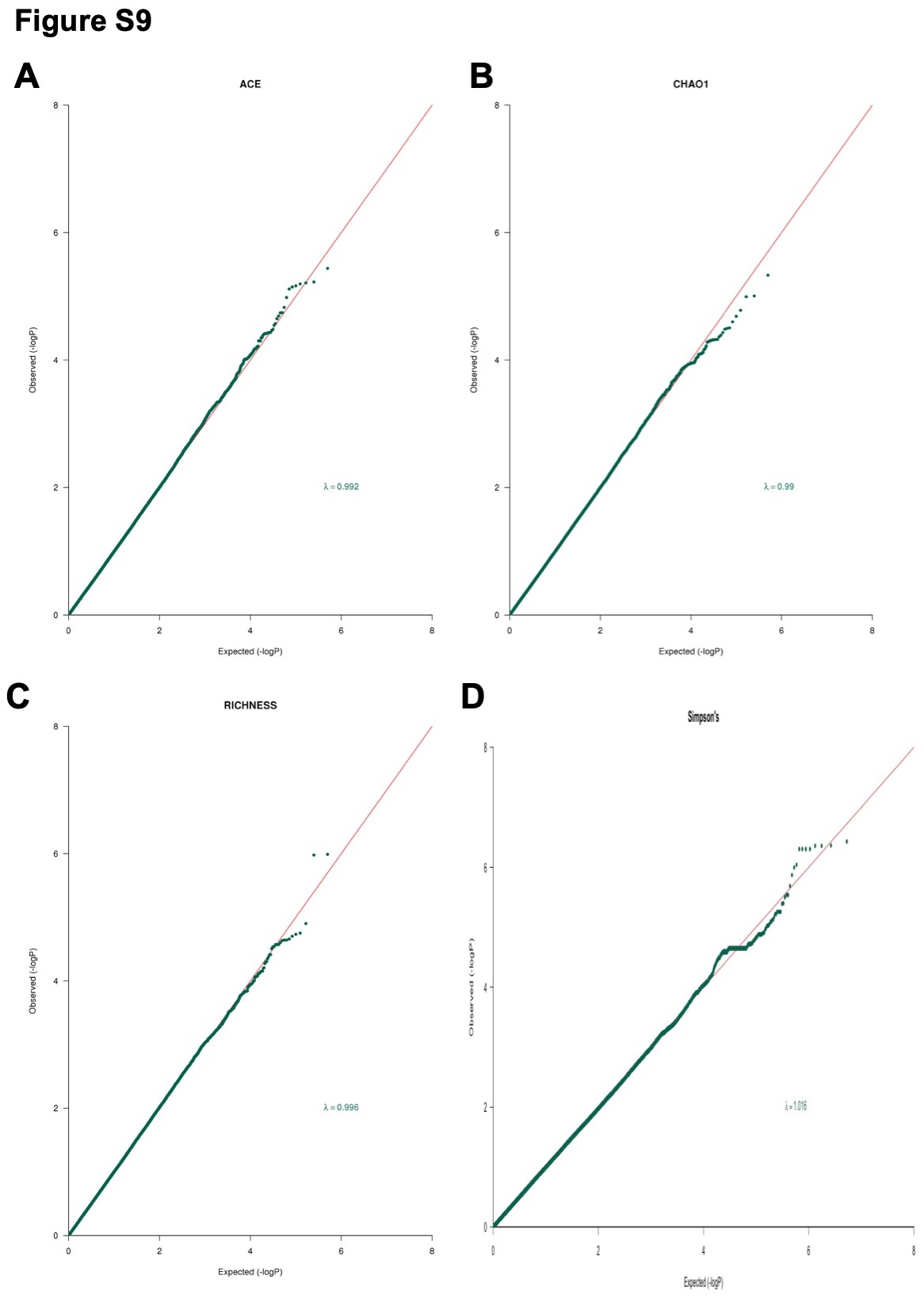
**

**Figure S9.** The quantile-quantile plots and lambda values of genome-wide associations preformed for (A) Simpson’s index, (B) Chao1 index, (C) richness and (D) ACE.

**
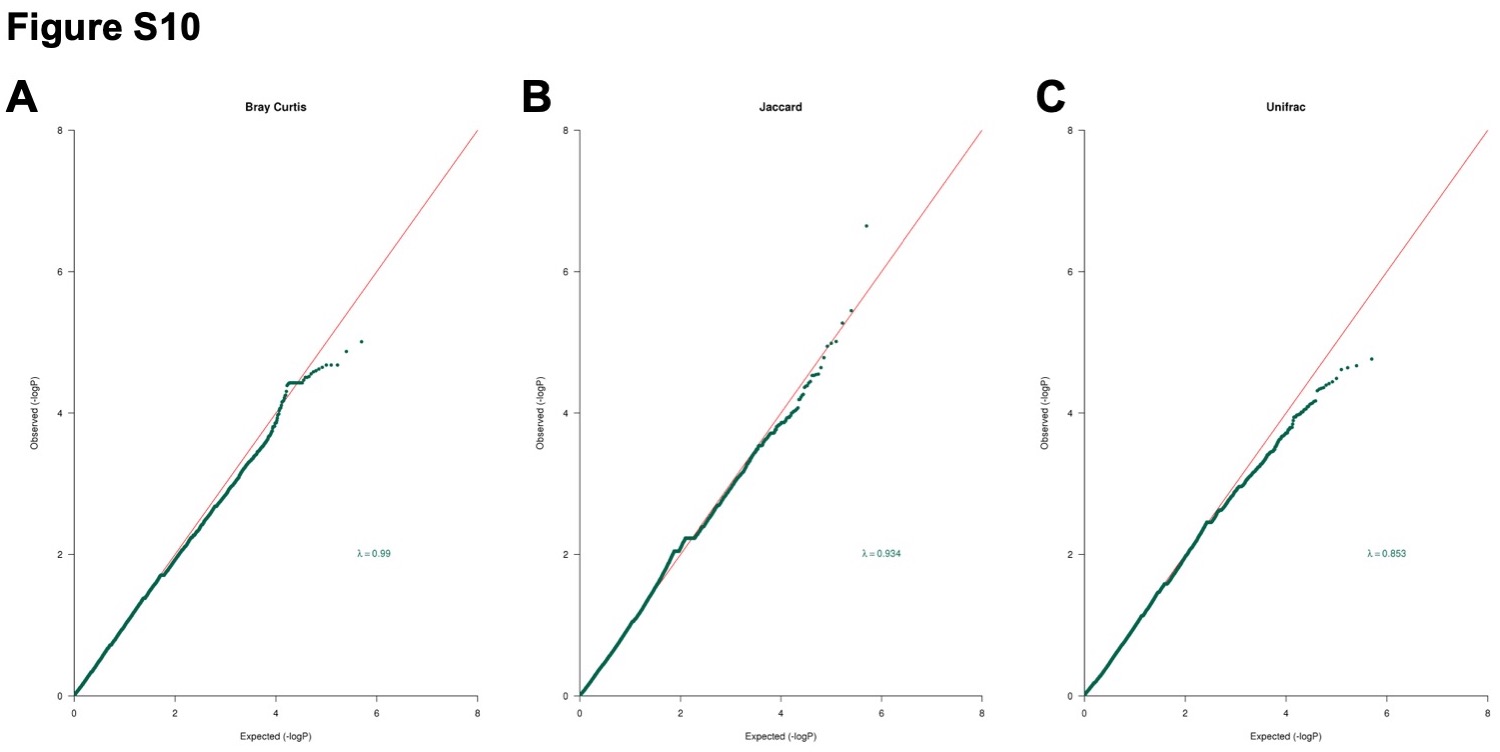
**

**Figure S10.** The quantile-quantile plots and lambda values of genome-wide associations preformed for (A) Bray Curtis, (B) Jaccard and (C) Unifrac distances.


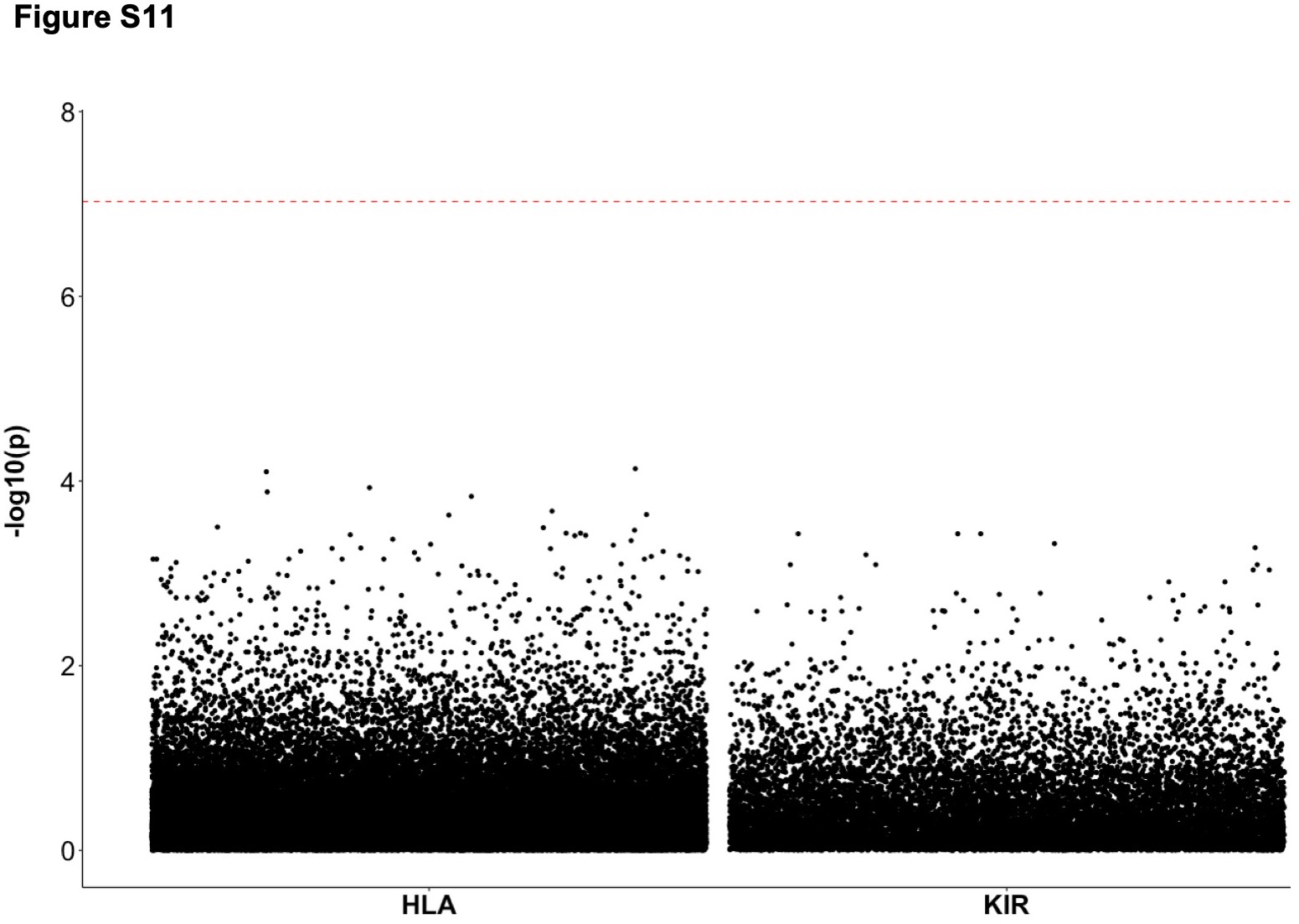


**Figure S11.** Manhattan plot of association results of all tested HLA and KIR alleles with all phenotypes (α-diversity, β-diversity, binary and quantitative taxa). The dashed horizontal line denotes significance threshold corrected for the number of tests performed (P_threshold_ < 9.42 x 10^-7^).

**
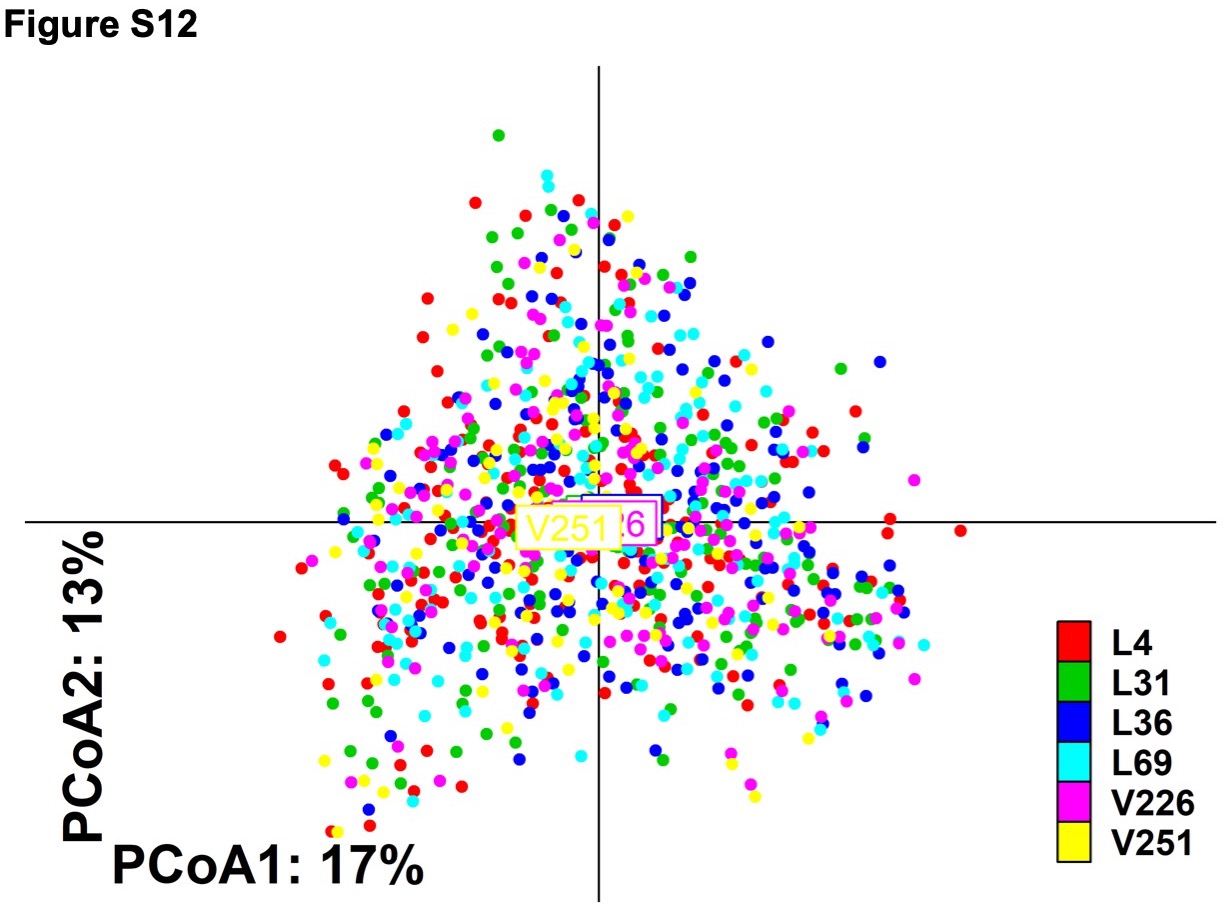
**

**Figure S12.** Principal Coordinate Analysis (PCoA) obtained from the genus level of MI samples deriving from different sequencing batches (indicated as L4, L31, L36, L69, V226, V251).

**
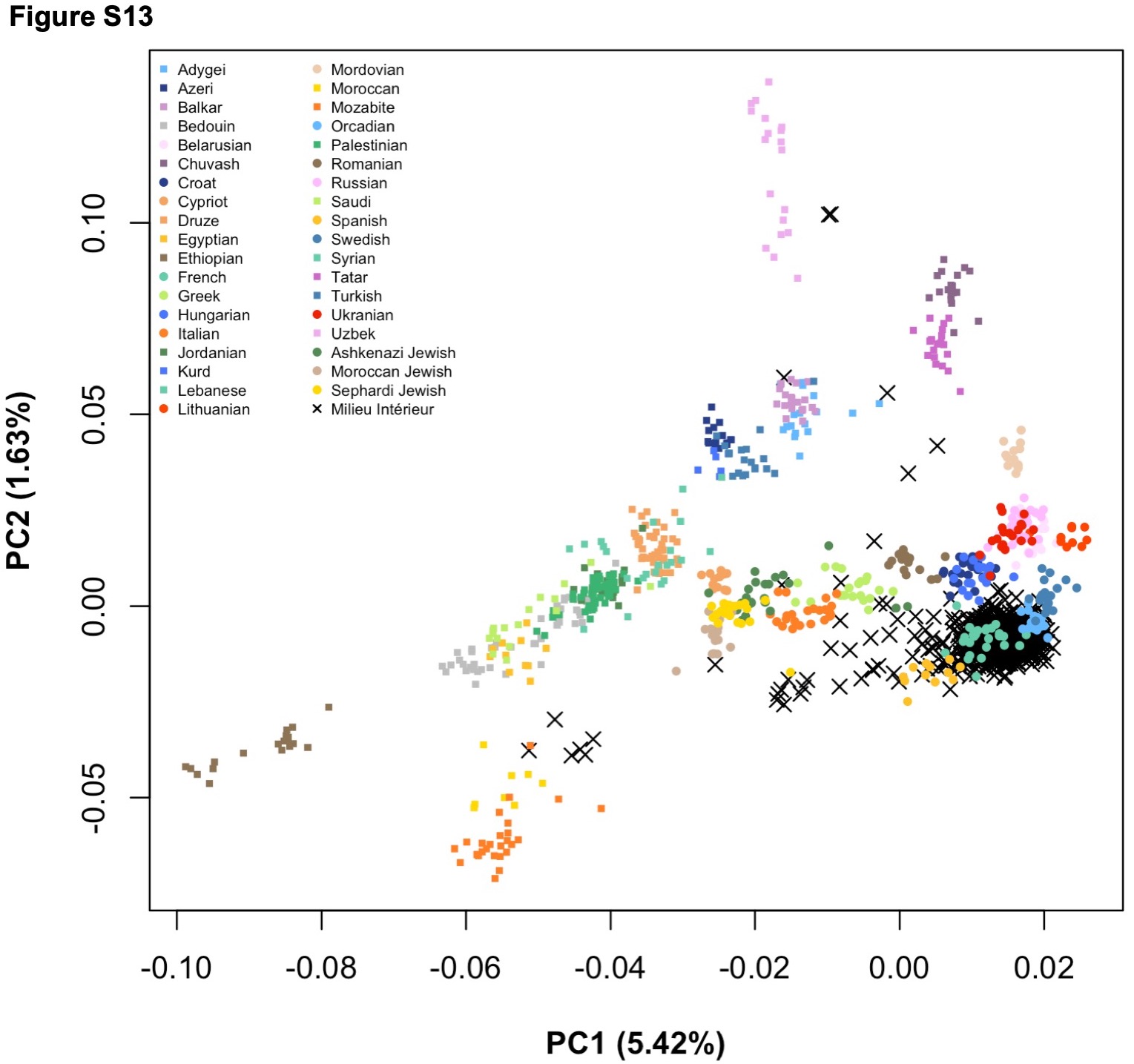
**

**Figure S13.** PCA plot of the genetic matrix data of MI donors. Adapted from [51].
